# Extracellular ATP activates store-operated Ca^2+^ entry in white adipocytes: functional evidence for STIM1 and ORAI1

**DOI:** 10.1042/BCJ20170484

**Published:** 2018-02-14

**Authors:** Mickaël F. El Hachmane, Anna Ermund, Cecilia Brännmark, Charlotta S. Olofsson

**Affiliations:** 1 Department of Physiology/Metabolic Physiology, Institute of Neuroscience and Physiology, University of Gothenburg, Göteborg, Sweden; 2 Department of CVMD Bioscience, AstraZenenca R&D Gothenburg, Gothenburg, Sweden; 3 Department of Medical Biochemistry and Cell Biology, Institute of Biochemistry, The Sahlgrenska Academy, University of Gothenburg, Göteborg, Sweden

**Keywords:** calcium imaging, Orai1, purinergic signalling, Stim1, store-operated calcium channels, white adipocyte

## Abstract

In the present study, we have applied ratiometric measurements of intracellular Ca^2+^ concentrations ([Ca^2+^]_i_) to show that extracellularly applied ATP (adenosine triphosphate) (100 µM) stimulates store-operated Ca^2+^ entry (SOCE) in 3T3-L1 adipocytes. ATP produced a rapid increase in [Ca^2+^]_i_ consisting of an initial transient elevation followed by a sustained elevated phase that could be observed only in the presence of extracellular Ca^2+^. Gene expression data and [Ca^2+^]_i_ recordings with uridine-5′-triphosphate or with the phospholipase C (PLC) inhibitor U73122 demonstrated the involvement of purinergic P2Y2 receptors and the PLC/inositol trisphosphate pathway. The [Ca^2+^]_i_ elevation produced by reintroduction of a Ca^2+^-containing intracellular solution to adipocytes exposed to ATP in the absence of Ca^2+^ was diminished by known SOCE antagonists. The chief molecular components of SOCE, the stromal interaction molecule 1 (STIM1) and the calcium release-activated calcium channel protein 1 (ORAI1), were detected at the mRNA and protein level. Moreover, SOCE was largely diminished in cells where STIM1 and/or ORAI1 had been silenced by small interfering (si)RNA. We conclude that extracellular ATP activates SOCE in white adipocytes, an effect predominantly mediated by STIM1 and ORAI1.

## Introduction

White adipose tissue (WAT) is an organ distributed at several locations in the body and with an essential role in the regulation of energy homeostasis. WAT is primarily composed of white adipocytes involved in the modulation of lipid metabolism as well as in the storage of fatty acids. Moreover, adipocytes have an endocrine role and release bioactive substances such as cytokines and hormone-like substances [1]. Adipose tissue dysfunctions are involved in metabolic disorders including type-2 diabetes and obesity [2].

Dynamic alterations of the intracellular Ca^2+^ concentration ([Ca^2+^]_i_) are vital to a wide array of cell biological processes such as signal transduction, muscle contraction, control of enzyme activity and regulation of exocytosis. However, cytoplasmic Ca^2+^ excess resulting from elevated Ca^2+^ influx, disturbances of intracellular Ca^2+^ sequestering and/or malfunctioning plasma membrane Ca^2+^ ATPases may cause cytotoxicity and trigger cell death. Thus, [Ca^2+^]_i_ needs to be tightly regulated [3,4]. Several pathways exist that result in an elevation of [Ca^2+^]_i_. In electrically excitable cell types, plasma membrane depolarization usually leads to activation of voltage-gated Ca^2+^ (Cav) channels allowing influx of Ca^2+^ from the extracellular space [5] and receptor activation may lead to release of Ca^2+^ from endoplasmic reticulum (ER) stores [3,4]. Store-operated Ca^2+^ entry (SOCE; also called capacitative calcium entry) is a major [Ca^2+^]_i_ elevating pathway present in most eukaryotic cells. In SOCE, depletion of intracellular Ca^2+^ ER stores leads to activation of plasma membrane-bound store-operated Ca^2+^ channels (SOCCs) and subsequent influx of Ca^2+^ from the extracellular space. Although the pathway by which depletion of intracellular calcium stores activates calcium entry was described 30 years ago [6], the main molecular players were identified in 2005 in the form of Stromal interaction molecule 1 (STIM1) [7,8]) situated in the ER membrane and the plasma membrane calcium release-activated calcium channel protein 1 (ORAI1) [9,10]. It is now known that intracellular Ca^2+^ store depletion translocates STIM1 to a region of ER located close to ORAI1 channels to allow protein–protein interaction. The contact leads to opening of ORAI1 channels with ensuing Ca^2+^ influx and the channels remain open until ER Ca^2+^ stores are refilled. SOCE is the main pathway of regulated Ca^2+^ influx in non-excitable cells and is usually caused by activation of hormone/neurotransmitter-regulated membrane-bound receptors [11]. The existence of STIM1 and ORAI1 has been confirmed in many non-excitable cell types [12] and STIM1 has been shown to participate in adipogenesis [13]. However, to the best of our knowledge, the functional existence of SOCCs has not been shown in white adipocytes.

Adenosine triphosphate (ATP) is not only a major energy source found within cells but also an important extracellular signalling molecule. ATP binds to plasma membrane-located purinergic receptors (P2Y or P2X), thus initiating intracellular signalling cascades. Many P2Y receptor subtypes have been identified in adipocytes and shown to be involved in adipocyte differentiation [14], leptin production and secretion [15,16] and in lipolysis [15]. Several P2Y receptors couple to activation of phospholipase C (PLC) with subsequent activation of inositol trisphosphate (IP_3_) receptors in the ER membrane leading to Ca^2+^ store depletion (reviewed in [17]). The activation of IP_3_ receptors has been proposed to be crucial for coupling between Ca^2+^ store emptying and activation of SOCCs [18].

Here, we have investigated SOCE and the functional existence of SOCCs in 3T3-L1 adipocytes. Dynamic measurements of [Ca^2+^]_i_ show that extracellularly applied ATP (100 µM) stimulates SOCE via interaction with P2Y2 receptors leading to activation of the PLC/IP_3_ pathway. The SOCE is inhibited by known SOCC antagonists as well as by siRNA (small interfering RNA) knockdown of STIM1 and/or ORAI1. Moreover, we confirm the presence of STIM1 and ORAI1 at the protein level.

## Materials and methods

### In vitro 3T3-L1 adipocyte differentiation

3T3-L1 cells were grown in a monolayer tissue culture flask maintained in a growth medium (DMEM with high-glucose, 4500 mg l^−1^; Life Technologies, Stockholm, Sweden). The medium was supplemented with 10% of heat-inactivated fetal bovine serum, 1% penicillin and streptomycin and 1% non-essential amino acids. The cells were placed in an incubator at 37°C with a humidified atmosphere of 95% oxygen and 5% carbon dioxide. After 2 or 3 days, when the cells were ∼70% confluent, they were split and resuspended in either 35 mm diameter plastic dishes (Nunc), 35 mm glass dishes (MatTek) or 12-well plates at a density of 1.5 × 10^5^ cells/ml, ready to be differentiated when a 90–100% confluence was obtained (∼2 days after seeding the cells). The first differentiation cocktail (D1) contained insulin (Sigma 170 µM), dexamethasone (Sigma; 10 mM) and 3-isobutyl-1-methylxanthine (Sigma, 5 mM). After 2 days, the medium was changed for a second differentiation cocktail (D2) containing 170 µM insulin only. The cells were used days 8 and 9 after the start of differentiation.

### Ratiometric calcium imaging

Cells were loaded for 90 min at room temperature (19–22°C) with Fura-2AM (2 µmol l^−1^; ThermoFisher) and pluronic acid (0.007% wt/vol; ThermoFisher). The bath medium consisted of External medium composed of 138 mM NaCl, 5.6 mM KCl, 2.6 mM CaCl_2_, 1.2 mM MgCl_2_, 5 mM HEPES and 5 mM Glucose (pH = 7.4) Changes in [Ca^2+^]_i_ were recorded by dual-wavelength microfluorimetry. Using minimal light, fura-2AM was excited at 340 and 380 nm by the use of a Lambda DG-4 filter system. Emitted light was collected above 510 nm. The data were recorded using MetaFluor software and calculated into absolute concentrations using equation 5 of [19]. All the data presented were collected at 32°C.

### Immunofluorescence

3T3-L1 adipocytes were grown and differentiated on 8-well chamber slides (Nunc® Lab-Tek® Chamber Slide™ system). Cells were fixed with 4% paraformaldehyde for 10 min at room temperature and then washed with Tris-buffered saline (TBS) pH 7.6 (composition in mM: 100 Trizma base, 15 NaCl). Unspecific binding was blocked with 3% donkey serum and the cells were permeabilized with 0.1% Triton X-100 in TBS for 1 h at room temperature. Primary antibodies made in the rabbit (Alomone Labs, Jerusalem, Israel) directed against ORAI1 (1 : 1000), STIM1 (1 : 500) or TRPC1 (1 : 100) were incubated over night at 4°C in blocking solution. Then, mouse monoclonal anti-Caveolin1 (Abcam, Cambridge, U.K.) antibody (1 : 500) was applied and incubated over night at 4°C. Donkey anti-rabbit Alexa 488 and donkey anti-mouse Alexa 647 secondary antibodies were diluted to 1 : 2000 in blocking solution and incubated for 1 h at room temperature. Nuclei were counterstained with Hoechst stain (Molecular Probes, ThermoFisher, Waltham, MA, U.S.A.). Images were acquired using a Zeiss LSM 700 upright confocal microscope (Carl Zeiss, Oberkochen, Germany) and analysed with the Imaris software (Bitplane, Zurich, Switzerland).

### Reverse transcription and quantitative polymerase chain reaction

We extracted and purified RNA using TRIzol (Life Technologies) and ReliaPrep™ RNA Cell Miniprep System (Promega). RNA quantity and purity was assessed using NanoDrop 1000 spectrophotometer. Total RNA was reverse-transcribed to cDNA by qScript Flex cDNA Kit (Quanta Biosciences) mixed primer strategy (random primers and oligo dT). SYBR Select Master Mix (Life Technologies) was used for quantitative PCR. The gene expression was normalized against β-actin (*Actb*) by application of the relative Δ*C*_t_ method (primer sequences are given in Supplementary Table S1). Primers were used at a concentration of 500 nM and PCR efficiencies were determined from the slope of the standard curve. Reactions without sample were used as a negative control.

### siRNA transfection

Cells were transfected with either Silencer® or Silencer® Select pre-designed siRNA (Ambion) at differentiation day 6 in Opti-MEM medium using 100 nM siRNA and lipofectamine 2000 (Life Technologies). siRNA identification: 64735 and s74488 [Stim1], s99510 and s99511 [Orai1], s75482 [Trpc1] and negative control #1 siRNA. Medium was replaced 8 h after transfection by antibiotic-free DMEM (10% FBS). The cells were used for experiments 60 h after the start of transfection procedure. Knockdown was validated by qPCR (quantitative polymerase chain reaction), and [Ca^2+^]_i_ was measured as described above.

### Data analysis

The statistical significance of variance between two means was calculated using Origin Pro8.5 (OriginLab Corporation, U.S.A.). Student's *t*-test, paired or unpaired as appropriate, was the statistical hypothesis test used unless otherwise specified. One-way ANOVA was applied when more than one group is compared with the same control. Data are presented as mean values ± SEM for the stated number of analysed cells (*n*) in a specific number of experiments (number in brackets). For the [Ca^2+^]_i_ imaging experiments, single cells were marked as a region of interest using the MetaFluor software. Confocal images were analysed for intensity over the plasma membrane in the green (TRPC1, ORAI1 and STIM1) and red channel (Caveolin1) using ImageJ. For each protein of interest, five images were analysed. Values were normalized to the highest value and presented as per cent of maximal intensity in each channel. Intensity profiles were mapped using GraphPad Prism 6.07.

## Results

### ATP elevates adipocyte intracellular calcium in a biphasic manner

**Figure 1.**
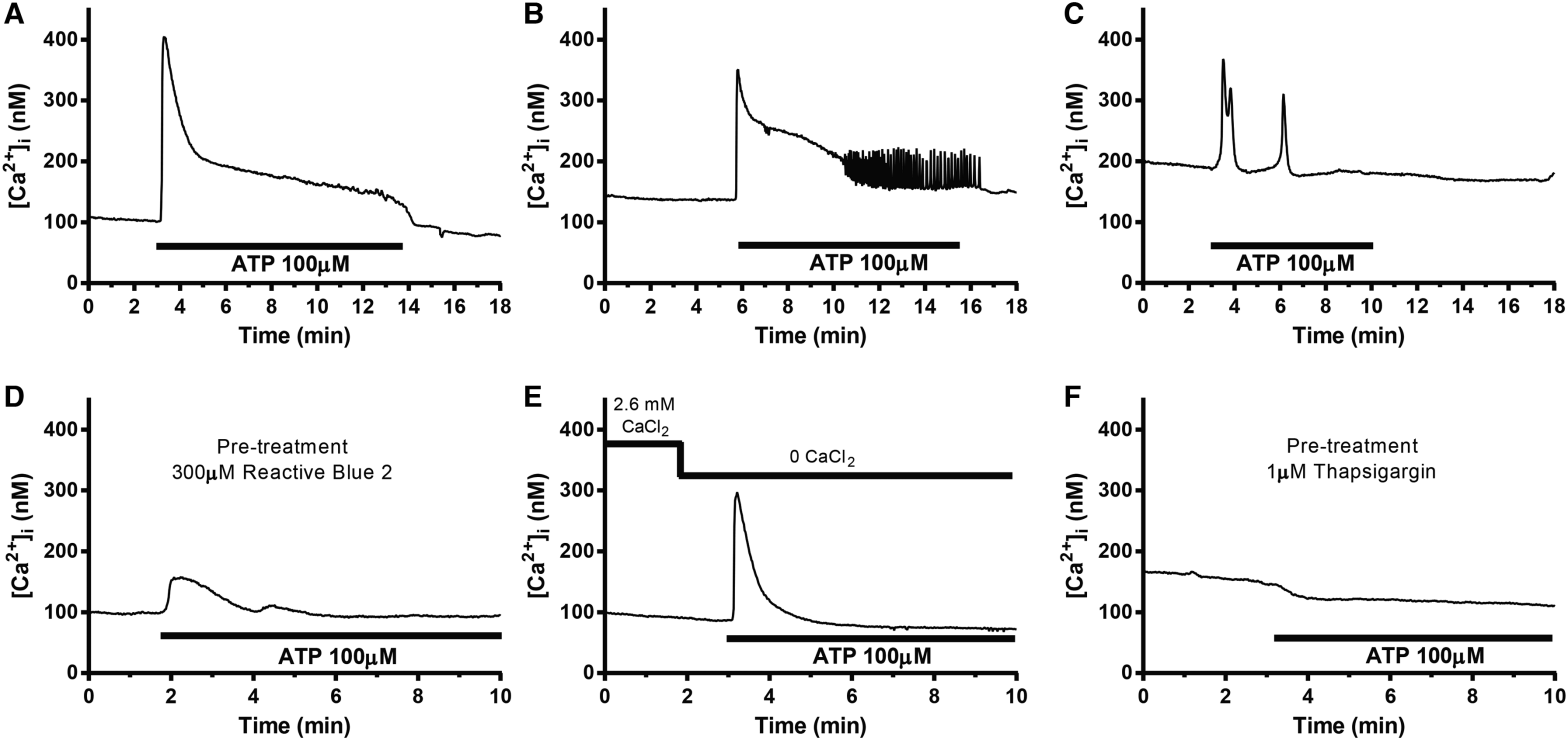
Representative traces showing effects of 100 µM ATP on 3T3-L1 adipocyte [Ca^2+^]_i_ under different conditions. (**A**–**C**) ATP responses in cells exposed to ATP in the presence of 2.6 mM extracellular Ca^2+^, *n* = 313 (10); (**D**) ATP added to cells pre-treated with 300 µM reactive blue 2, *n* = 124 (3); or (**E**) in the absence of extracellular Ca^2+^, *n* = 114 (3); (**F**) ATP applied to cells pre-treated with 1 µM thapsigargin, *n* = 142 (5). Number of separate experiments indicated in brackets; *n* represents number of analysed cells.

We investigated the effect of extracellularly applied ATP on 3T3-L1 adipocyte [Ca^2+^]_i_ (Figure 1A–F). Application of 100 µM ATP generated a rapid rise in [Ca^2+^]_i_ consisting of an initial transient increase followed by a sustained phase during which [Ca^2+^]_i_ remained elevated for as long as ATP was present (Figure 1A). The fast-transient increase was seen in all studied cells. The sustained elevation was present in 73% of the adipocytes (Figure 1A), whereas in 20% of the cells the sustained plateau was replaced by rhythmic transient rises of [Ca^2+^]_i_ originating from an elevated plateau level (Figure 1B). In the remaining 7% of cells, ATP application produced one or several [Ca^2+^]_i_ peaks originating from a Ca^2+^ level similar to that before application of the nucleotide (Figure 1C). Pre-treatment with the P2Y receptor antagonist reactive blue 2 (RB-2; 300 µM for 30 min) inhibited the initial transient increase by 70% (measured as Δpeak [Ca^2+^]_i_ increase) from 186 ± 4 nM upon application of ATP alone (Figure 1A–C) to 58 ± 4 nM in RB-2 pre-treated cells (Figure 1D; *P* < 0.001 vs. ATP alone). The second phase was abolished in RB-2 exposed adipocytes.

To determine the origin of the calcium involved in the ATP-induced cytoplasmic calcium increase, the cell dish was perfused during the recordings with a solution lacking Ca^2+^. The initial [Ca^2+^]_i_ transient was inhibited by 30% (Δpeak [Ca^2+^]_i_; 129 ± 5 nM vs. the Δpeak value of 186 ± 4 nM in the presence of 2.6 mM extracellular Ca^2+^; *P* < 0.001) and the stable elevation was abolished in the absence of extracellular Ca^2+^ (Figure 1E). To further explore the origin of Ca^2+^, cells were treated with the sarco/endoplasmic reticulum Ca^2+^-ATPase (SERCA) inhibitor thapsigargin (1 µM during 30 min) to deplete intracellular calcium stores. The ATP response was absent from thapsigargin pre-treated cells (Figure 1F). Interestingly, addition of ATP to thapsigargin pre-treated cells, in fact, resulted in a decrease in [Ca^2+^]_i_ (Δchange = 20 nM; *P* < 0.001). This decrease is likely due to ATP-dependent activation of plasma membrane Ca^2+^ pumps [20,21].

Our results suggest that ATP triggers release of Ca^2+^ from intracellular stores via activation of P2Y receptors generating the first transient [Ca^2+^]_i_ peak. The store depletion subsequently activates influx of Ca^2+^ from the extracellular space, observed as a stable [Ca^2+^]_i_ elevation.

### ATP triggers calcium store depletion via stimulation of P2Y2 receptors and activation of a PLC/IP_3_-dependent pathway

**Figure 2.**
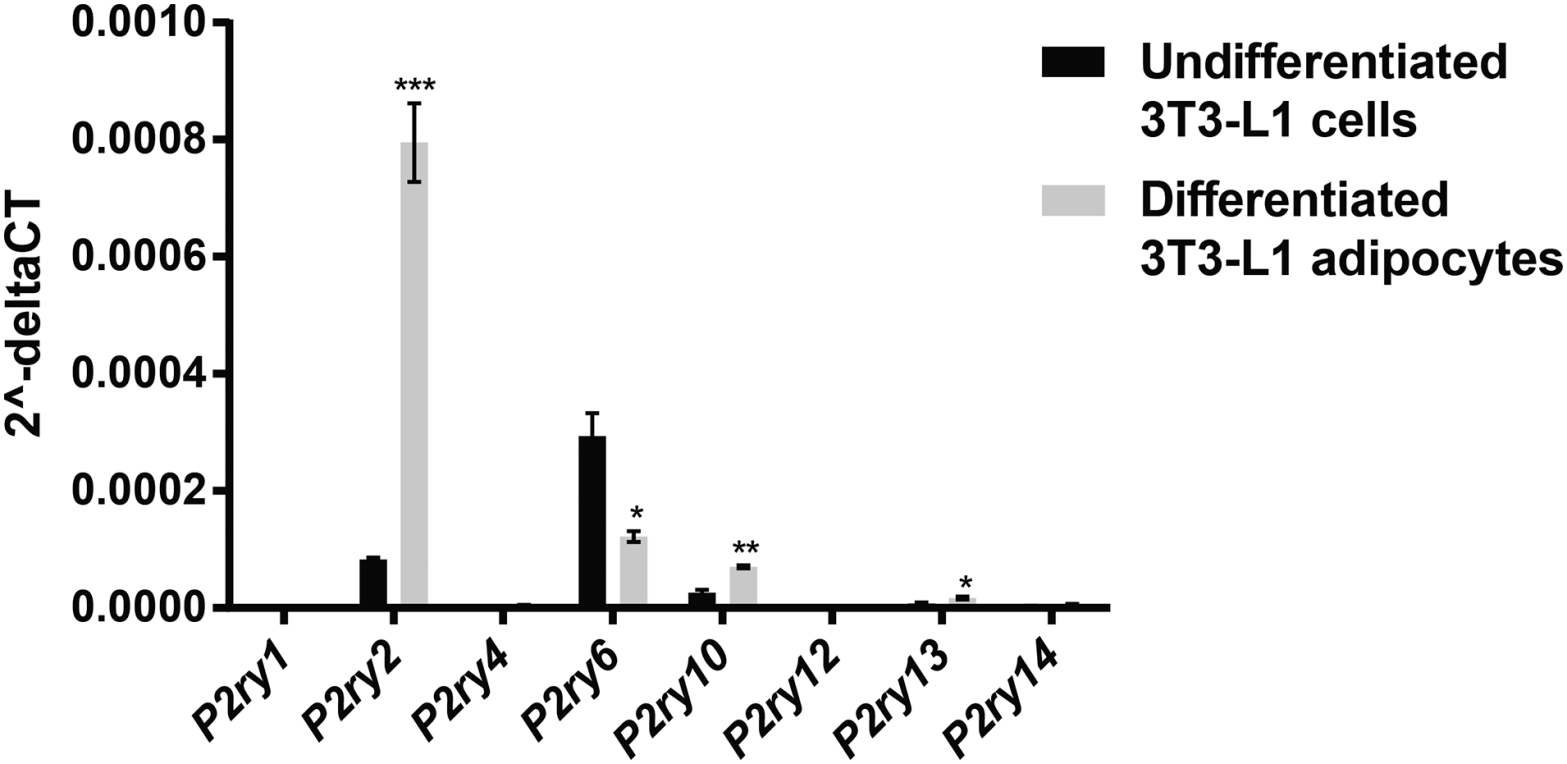
Gene expression of P2Y receptors in undifferentiated and differentiated 3T3-L1 adipocytes. The mRNA levels are normalized against its respective endogenous β-actin mRNA level and expressed as $2^{-\Delta C_{t}}$ values. *P < 0.05; **P < 0.01; ***P < 0.001. Results from three separate experiments.

**Figure 3.**
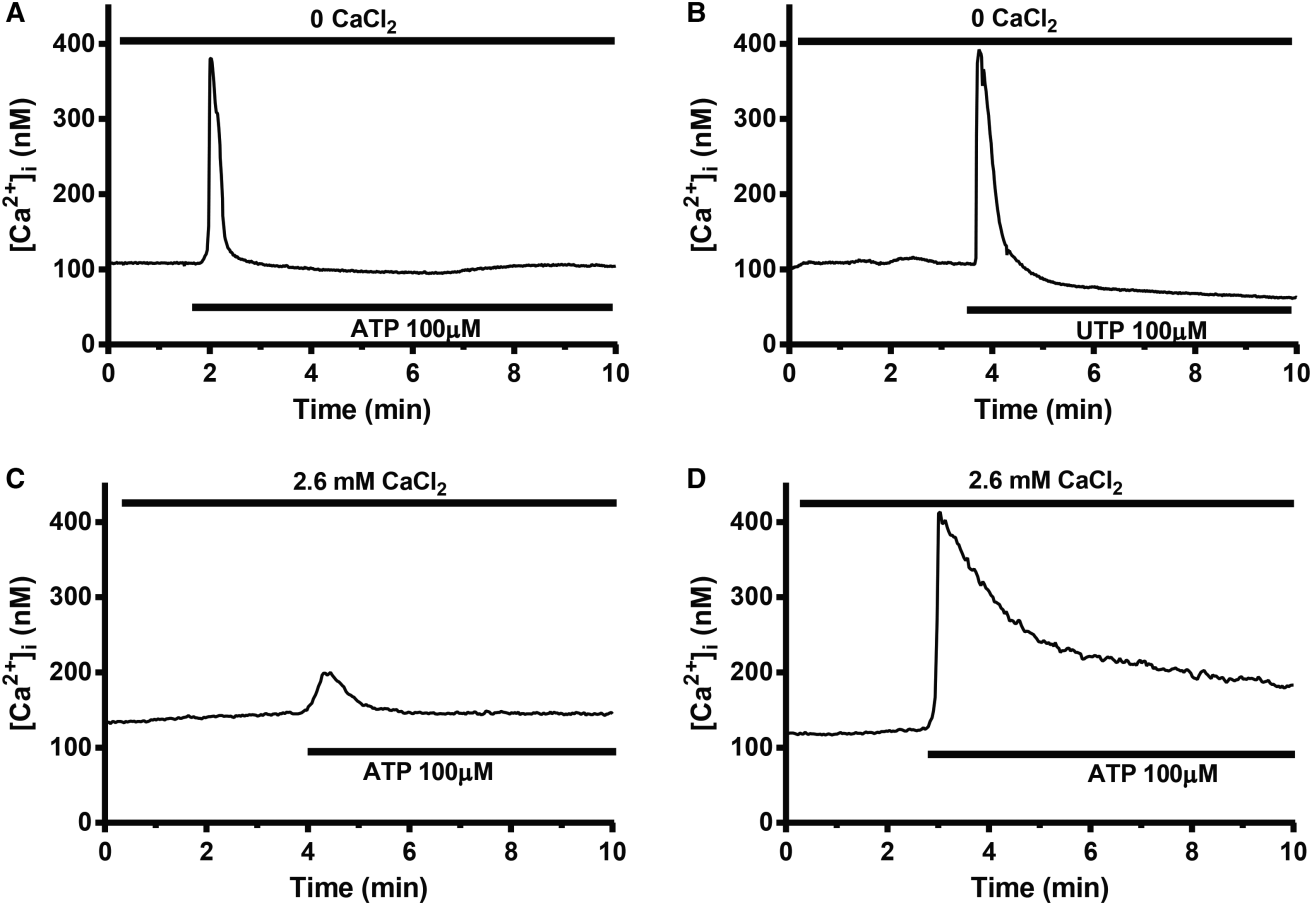
Representative traces showing effects of ATP, UTP or the PLC inhibitor U73122 on 3T3-L1 adipocyte [Ca^2+^]_i_ under different conditions. (A) 100 µM ATP or (B) 100 µM UTP was added to cells in the absence of extracellular Ca^2+^. (C) Cells were pre-treated with 10 µM PLC inhibitor U73122 during 30 min prior to the start of the recording. (D) Adipocytes were correspondingly pre-exposed to the ineffective analogue U73343. [Ca^2+^]_i_ was analysed in 157 (4) (A), 204 (5) (B), 182 (6) (C) and 102 (4) (D) separate cells (number in brackets indicates number of separate recordings).

We investigated the gene expression of P2Y receptors (the P2ry gene family) in undifferentiated and differentiated 3T3-L1 adipocytes (Figure 2). In agreement with previous findings, the P2ry2 gene was abundantly expressed in differentiated adipocytes. P2ry6 and P2ry10 were also detected but at significantly lower levels. Furthermore, P2Y6 and P2Y10 receptors are activated by UDP [22] and lysophosphatidic acid [23], respectively, and not by ATP. In order to functionally define the presence of P2Y2 receptors, we stimulated the adipocytes with uridine-5′-triphosphate (UTP). P2Y2 is the only known purinergic receptor activated by both UTP and ATP [22]. As shown in Figure 3A,B, UTP application resulted in an [Ca^2+^]_i_ peak of a similar magnitude as that produced by ATP; Δpeak values were 199 ± 10 and 198 ± 9 nM, respectively.

P2RY2 receptors are coupled to the G protein Gq11 and trigger [Ca^2+^]_i_ increases via the PLC/IP_3_ pathway. IP_3_ activates ER-localized IP_3_ receptors that work as Ca^2+^ channels thus leading to depletion of ER Ca^2+^ stores [24]. As shown in Figure 3C, pre-treatment with the PLC inhibitor U73122 (10 µM during 30 min) reduced the ATP-induced transient calcium increase by 75% (Δpeak [Ca^2+^]_i_ averaged 47 ± 5 nM) and inhibited the sustained [Ca^2+^]_i_ elevation. ATP remained capable of elevating [Ca^2+^]_i_ in adipocytes pre-treated with U73343, a structurally close analogue to U73122 with no demonstrated effect on PLC. The Δpeak [Ca^2+^]_i_ elevation produced in U73343-exposed adipocytes averaged 171 ± 15 nM (Figure 3D), a magnitude similar to that attained with ATP in non-treated cells (cf. Figure 1A).

Our results propose that ATP signals via P2Y2/Gq11/PLC to stimulate IP_3_ receptor-mediated Ca^2+^ store depletion which, in turn, activates influx of Ca^2+^ over the plasma membrane.

### The sustained [Ca^2+^]_i_ elevation is blocked by the SOCE inhibitors 2-APB, SKF96365 and Gd^3+^

**Figure 4.**
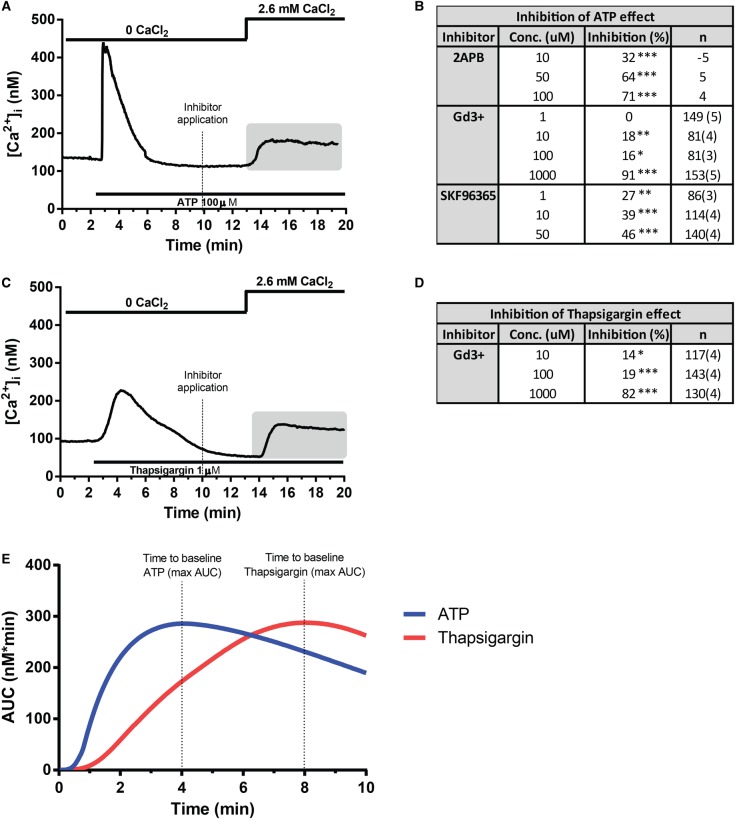
Effects of the SOCE inhibitors 2-APB, SKF96365 and Gd^3+^ on the sustained [Ca^2+^]_i_ elevation produced by 100 µM ATP or by 1 µM thapsigargin. Representative traces are shown for ATP (A) and for thapsigargin (C). The grey shaded areas indicate the part of the experiments where the effects of the SOCE inhibitors on reintroduction of Ca^2+^ were analysed. The inhibitory effects compared with control are summarized in (B) for ATP and in (D) for thapsigargin. (E) The amount of Ca^2+^ released from ER stores by ATP or thapsigargin exposure analysed as the average area under the curve (AUC; nM min). Effect of the different drugs applied was analysed using one-way ANOVA with Tukey *post hoc* test. *P < 0.05; **P < 0.01; ***P < 0.001.

We hypothesized that ER Ca^2+^ store depletion activates SOCCs in the adipocyte plasma membrane. To confirm that SOCE underlies the stable [Ca^2+^]_i_ elevation, we investigated the effect of the SOCE inhibitors 2-APB, SKF96365 and Gd^3+^. As shown in Figure 4A, ATP was applied to 3T3-L1 adipocytes in a Ca^2+^-free extracellular solution. ATP again stimulated the transient [Ca^2+^]_i_ increase after which [Ca^2+^]_i_ declined to stabilize at a baseline level similar to that measured before ATP application. The SOCE inhibitors were subsequently added separately to the Ca^2+^-free solution at different concentrations, then 3 min later 2.6 mM Ca^2+^ was perfused in the continued presence of the inhibitor. Under control conditions, the increase in [Ca^2+^]_i_ caused by the Ca^2+^ reintroduction averaged 56 ± 2 nM (206 analysed cells in seven separate experiments). 2-APB, SKF96365 and Gd^3+^ all inhibited the [Ca^2+^]_i_ increase observed upon reintroduction of Ca^2+^. These effects occurred in a concentration-dependent manner (Figure 4B).

We next tested the acute addition of thapsigargin to deplete the Ca^2+^ stores (Figure 4C). The inhibition of SERCA by this compound is well known to induce SOCE [25]. Thapsigargin (1 µM applied together with a Ca^2+^-depleted solution) induced a Δpeak [Ca^2+^]_i_ of 95 ± 4 nM (*n* = 97), a significantly lower magnitude than that produced by ATP under the same conditions (280 ± 7 nM; *n* = 208 *P* < 0.001). This slower response is because thapsigargin inhibition of ER Ca^2+^ refilling results in gradual Ca^2+^ leakage, whereas ATP induces rapid Ca^2+^ depletion via activation of IP_3_ receptors [24]. As shown in Figure 4E, analysis of the area under the curve showed that a similar amount of Ca^2+^ was released under both conditions (337 ± 12 nM min with thapsigargin and 361 ± 17 nM min with ATP). The time to [Ca^2+^]_i_ peak and the reversal to [Ca^2+^]_i_ baseline were largely delayed upon addition of thapsigargin when compared with ATP ([Ca^2+^]_i_ peak observed at 3.4 ± 0.3 min upon addition of thapsigargin and at 0.33 ± 0.02 min in response to ATP). Reintroduction of Ca^2+^ elevated [Ca^2+^]_i_ of a magnitude comparable to that measured in the ATP experiments (Δplateau 55 ± 2 nM, *n* = 152; *P* = 0.7 vs. Δplateau value with ATP). In keeping with the ATP experiments, Gd^3+^ inhibited the elevation caused by the reintroduction of Ca^2+^ in a dose-dependent fashion, thus resembling that observed when Ca^2+^ stores were depleted by ATP (Figure 4D).

### The key SOCE signalling proteins STIM1, ORAI1 and TRPC1 are present in 3T3-L1 adipocytes

**Figure 5.**
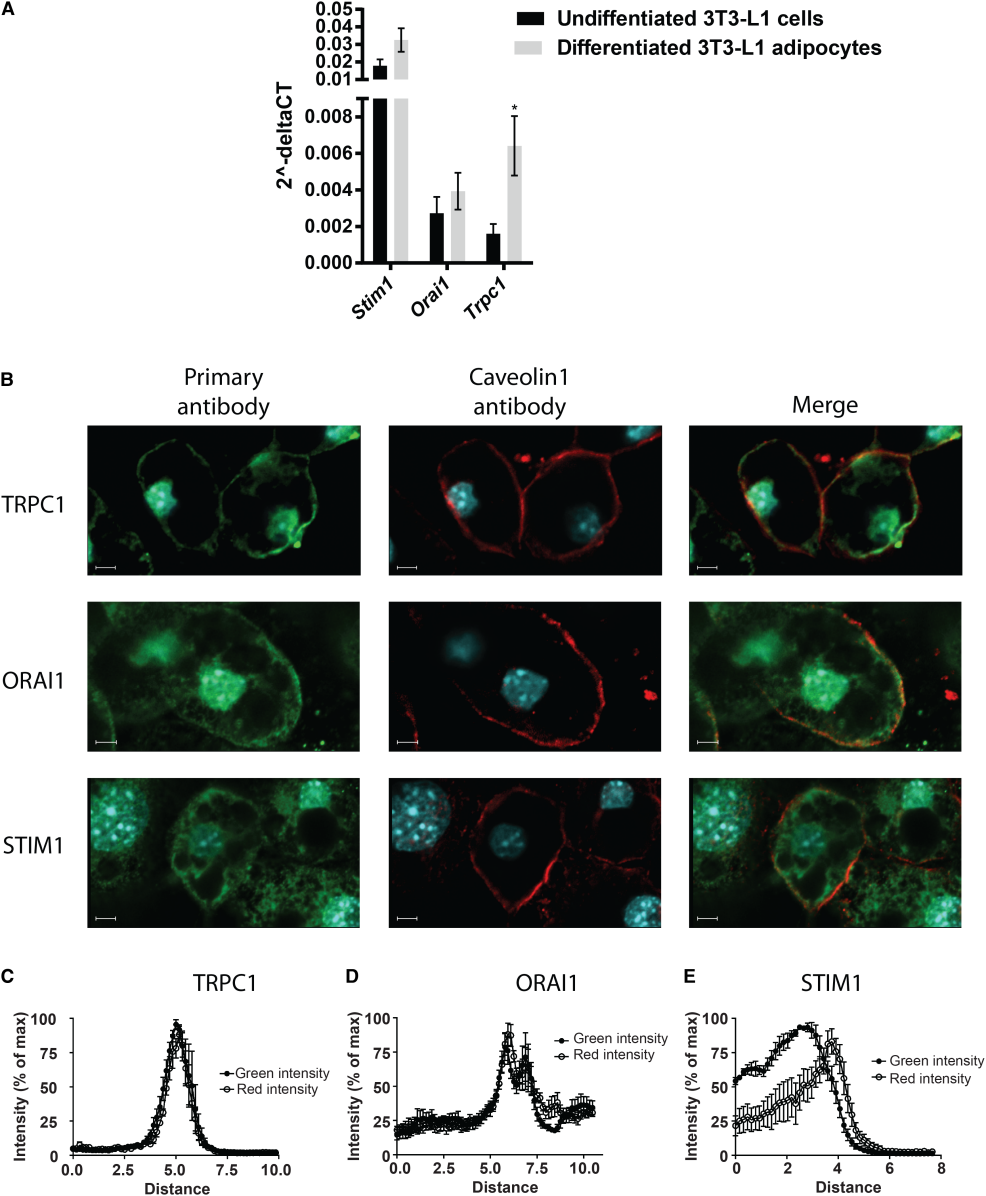
The presence of STIM1, ORAI1 and TRPC1 in 3T3-L1 adipocytes. (**A**) mRNA levels of *Stim1*, *Orai1* and *Trpc1*. Gene-specific mRNA levels are normalized against its respective β-actin mRNA level; (**B**) representative confocal images of adipocytes immunostained for TRPC1, ORAI1 and STIM1; (**C**–**E**) quantification of fluorescence intensity in adipocytes stained for (**C**) TRPC1, (**D**) ORAI1 and (**E**) STIM1 in five confocal images. The intensity is presented with the fluorescence intensity of Caveolin1 immunostaining in the same samples to show the intensity peak of the proteins of interest in relation to the plasma membrane (Caveolin1). *P < 0.05.

It is widely agreed that STIM1 is the sensor that conveys the Ca^2+^ store depletion to plasma membrane SOCCs and that ORAI1 is the main Ca^2+^ channel activated by STIM1 [11]. However, substantial evidence exists to support the participation of additional SOCCs, but the identity of other contributing channels and how they interact with ORAI1 and each other is, to say the least, unclear. Transient receptor potential (TRP) channels compose of a large family of Ca^2+^ permeable channels with TRPC channels proposed to participate in SOCE [26]. Many recent studies in particular suggest TRPC1 as a key component in SOCE ([27–29] and reviewed in [26]) and its presence in adipocytes has been shown [30]. We thus investigated the gene expression of *Stim1*, *Orai1* and *Trpc1* in 3T3-L1 adipocytes. As shown in Figure 5A, all three genes were expressed. We performed immunocytochemistry in order to verify the translation of gene transcripts into proteins. Figure 5B shows confocal images of 3T3-L1 adipocytes stained with antibodies against STIM1, ORAI1 and TRPC1 (antibody against Caveolin1 used as plasma membrane marker). The three proteins were clearly expressed and quantification of fluorescence intensities of the proteins of interest and Caveolin1 showed that the two SOCCs were notably membrane associated, while STIM1 was more internally localized (Figure 5C–E).

### The sustained [Ca^2+^]_i_ elevation is blocked by siRNA knockdown of *Stim1* or *Orai1*

**Figure 6.**
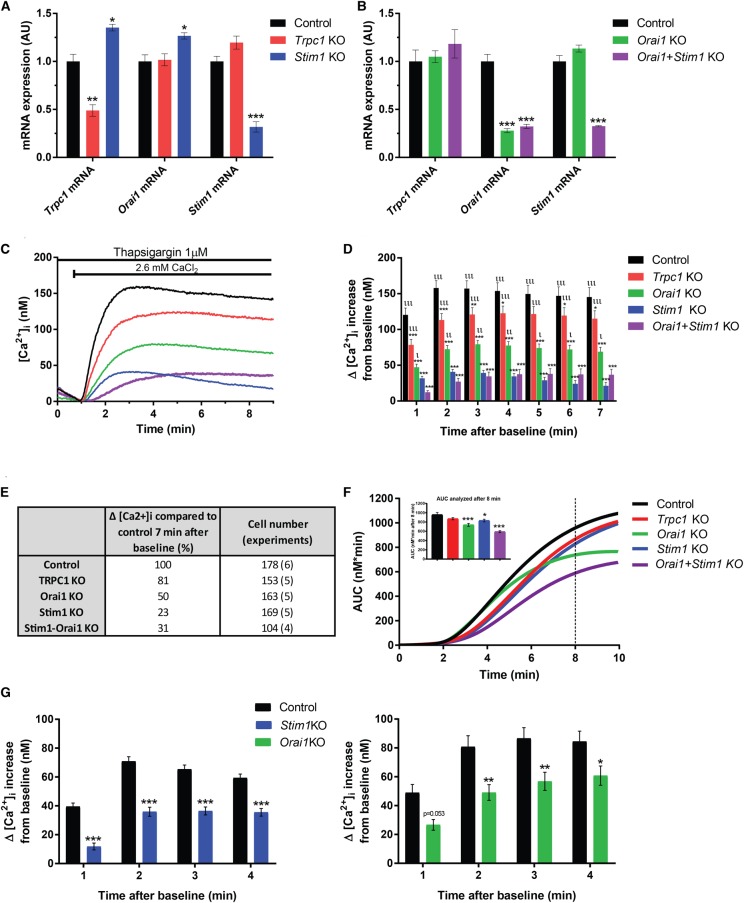
siRNA knockdown of *Stim1*, *Orai1* and *Trpc1* and gene silencing effects on SOCE. (**A** and **B**) mRNA levels for *Stim1*, *Orai1* and *Trpc1* upon knockdown (KO) of each gene separately as well as upon the simultaneous silencing of *Stim1* and *Orai1*; (**C**) traces of average [Ca^2+^]_i_ in response to an elevation of extracellular Ca^2+^ from 0 to 2.6 mM. Cells were treated with thapsigargin (1 µM) during 10 min prior to the reintroduction of a Ca^2+^-containing solution (not shown); (**D**) Average Δpeak [Ca^2+^]_i_ increase at different time points under the conditions shown in **C**. (**E**) Percentage remaining Δpeak [Ca^2+^]_i_ at 7 min under the different KO conditions compared with control (set to 100%). The difference in [Ca^2+^]_i_ levels was analysed by two-way repeated measure ANOVA with Dunnett's multiple comparisons test at each time point. *P < 0.5, **P < 0.01; ***P < 0.001 vs. control (Ctrl); ι *P* < 0.5; ιι *P* < 0.01; ιιι *P* < 0.001 vs. STIM1-ORAI1 KO. (**F**) Amount of Ca^2+^ released from ER stores by thapsigargin exposure analysed as the average area under the curve (AUC; nM min). Inserted histogram shows difference at time point 8 min analysed by one-way ANOVA with Bonferroni's multiple comparisons test (*P < 0.05, ***P < 0.001 vs. control). (**G**) Gene silencing of *Stim1* and *Orai1* using different sequences and effects of siRNA knockdown on SOCE. Average Δpeak [Ca^2+^]_i_ increase at different time points in response to an elevation of extracellular Ca^2+^ from 0 to 2.6 mM upon knockdown of Stim1 (left) or Orai1 (right). The difference in [Ca^2+^]_i_ levels was analysed by two-way repeated measure ANOVA with Bonferroni's multiple comparisons test at each time point. *P < 0.05, **P < 0.01; ***P < 0.001 vs. control.

The pharmacology of SOCC inhibitors is complex and they are known to affect targets other than those involved in SOCE [31]. Thus, to accurately confirm the functional existence of SOCE and its key molecular constituents, 3T3-L1 adipocytes were transfected with siRNA against *Stim1* or *Orai1* (alone or in combination) or with a scramble control. Owing to the suggested role of TRPC1 in SOCE, we also tested the effect of *Trpc1* knockdown. As shown in Figure 6A,B, siRNA transfection reduced the expression of *Trpc1* by ∼50% and that of *Stim1* and *Orai1* by ∼70% compared with the scramble control. The reduced expression of *Stim1* resulted in a slight up-regulation of *Trpc1* and *Orai1* mRNA levels (Figure 6A). We measured [Ca^2+^]_i_ in siRNA-transfected cells exposed to thapsigargin in the absence of Ca^2+^ and analysed the increase generated by reintroduction of a Ca^2+^-containing solution (same protocol as in Figure 3). As shown in Figure 6C,D, silencing of *Stim1* alone or in combination with *Orai1* potently inhibited the [Ca^2+^]_i_ elevation triggered by wash-in of 2.6 mM Ca^2+^, at all time points investigated. Single knockdown of *Orai1* also inhibited the [Ca^2+^]_i_ increase rather effectively, although to a significantly smaller extent than that produced by the combined silencing of *Stim1* and *Orai1*. In agreement with the proposed participation of TRPC1 in SOCE, knockdown of this channel led to a slight but significant reduction in the [Ca^2+^]_i_ increase. To investigate if knockdown of the target genes affected ER Ca^2+^ content and/or the ability to mobilize Ca^2+^, we analysed the area under the curve during thapsigargin addition. As may be anticipated, knockdown of *Stim1* or *Orai1*, alone or together, reduced the amount of Ca^2+^ mobilized by thapsigargin (Figure 6F). Surprisingly, the amount of Ca^2+^ mobilized under control conditions was significantly larger than in the experiments with thapsigargin in Figure 4C. Moreover, the magnitude of Ca^2+^ influx upon reintroduction of extracellular Ca^2+^ was notably larger than in the previous experiments using the SERCA inhibitor (157 ± 13 nM in Figure 6D vs. 55 ± 2 nM in Figure 4). The above differences could be due to either cell variation or to that siRNA transfection as such affects the Ca^2+^ stores. To verify that the functional changes seen in the siRNA-transfected cells were related to knockdown of the specific genes, 3T3-L1 adipocytes were transfected with other siRNA sequences against *Stim1* or *Orai1* (see Materials and methods). Transfection with the alternative siRNA sequences reduced the expression of *Stim1* by 55% and that of *Orai1* by 65% compared with the scramble control (not shown). We again measured the increase in [Ca^2+^]_i_ upon reintroduction of Ca^2+^ to thapsigargin-exposed cells. As shown in Figure 6G, the [Ca^2+^]_i_ elevation was, in agreement with data in Figure 6C,D, diminished in adipocytes transfected with *Stim1* or *Orai1* siRNA compared with scramble siRNA controls. Notably, the magnitude of maximum Ca^2+^ influx upon reintroduction of extracellular Ca^2+^ (∼80 nM) was in this experimental series in a range between that measured in Figures 4 and 6D, thus reinforcing that cell variability rather than the siRNA transfection itself underlies the variations in Ca^2+^ storage/dynamics. In conclusion, our knockdown experiments confirm the presence of SOCE in white adipocytes and propose that STIM1 and ORAI1 are the chief components.

## Discussion

The findings described here demonstrate the functional existence of SOCE in white adipocytes. We present evidence of the presence of the two main molecular players involved in SOCE: the ER-localized calcium sensor STIM1 and the plasma membrane Ca^2+^ permeable channel ORAI1 and we show the regulation of SOCE by external ATP. Ca^2+^ entry via store-operated channels has previously been shown in pre-adipocytes [32] and STIM1-mediated Ca^2+^ influx has also been demonstrated to negatively affect pre-adipocyte differentiation and adipogenesis [13]. However, our study is, to the best of our knowledge, the first to demonstrate the functional occurrence of STIM1 and ORAI1 as well as the stimulation of SOCE via purinergic receptors in lipid-filled adipocytes. Below we confer some possible implications of our findings.

### Origin and physiological role of external ATP

In addition to its many roles in intracellular signalling, ATP also acts as an extracellular signalling molecule via interaction with purinergic receptors. Here, we show, in line with a previous study [15], that ATP elevates adipocyte [Ca^2+^]_i_ via binding to P2Y2 receptors and activation of the PLC/IP_3_ pathway. WAT is abundantly innervated by the sympathetic nervous system and adrenergic nerve terminals co-release ATP with norepinephrine [33–35]. As a consequence, ATP is released in very close vicinity to the adipocytes upon sympathetic stimulation. Moreover, neuroendocrine cell secretory vesicles typically contain large quantities of ATP and both chromaffin cell catecholamine-containing vesicles [36,37] and pancreatic β-cell insulin granules [38,39] have been shown to contain substantial amounts of ATP. The white adipocyte is now known to be an endocrine cell [1] and adipocyte secretory hormone-containing vesicles can be envisaged to also accommodate ATP. ATP released from sympathetic nerves or from the adipocyte itself may act in an auto- and paracrine fashion.

Extracellular ATP activation of purinergic receptors induces elevations of [Ca^2+^]_i_ in several cell types [22,40] including white adipocytes [15,16,41]. The ATP-induced elevation of adipocyte [Ca^2+^]_i_ has been shown to involve activation of both P2Y1 [16] and P2Y2 receptors [15]. We were, however, unable to detect expression of *P2ry1* in 3T3-L1 adipocytes (Figure 2). The *P2ry* mRNA levels (Figure 2) together with our findings of UTP-induced [Ca^2+^]_i_ elevations (Figure 3B) suggest that ATP activation of P2Y2 receptors may have a key role in the regulation of Ca^2+^-dependent processes in the white adipocyte.

### Ca^2+^-dependence of adipocyte metabolic processes

Ca^2+^ has been proposed to affect many processes, such as lipolysis, secretion of adipokines and glucose uptake, in the white adipocyte . The role of Ca^2+^ in lipolysis (the breakdown of stored lipids into glycerol and fatty acids) is not fully determined. Ca^2+^ has been shown to enhance catecholamine-/cAMP-stimulated lipolysis in rats [42–44]. In contrast, a study in human adipocytes instead shows an inhibitory effect of Ca^2+^ on isoprenaline-induced lipolysis [45]. A recent investigation proposes a role of SOCE in lipolysis and lipid metabolism. However, this study lacks experimental data from mature (lipid-filled) adipocytes [46].

White adipocytes secrete a multitude of biologically active substances commonly referred to as adipokines [1]. The hormones leptin and adiponectin have, in particular, been extensively studied due to their important roles in the regulation of whole-body metabolism and because circulating levels of those adipokines are altered in obesity [47]. Ca^2+^ has important roles in the regulation of the secretion of both leptin and adiponectin, although not as a trigger of release as is the typical role of Ca^2+^ in other endocrine cell types [48]. Nonetheless, Ca^2+^ has been shown to be crucial for insulin-stimulated leptin secretion [49–52]. The Ca^2+^ seems to, at least in part, come from the extracellular space since incubation of adipocytes with a Ca^2+^-depleted medium markedly repressed insulin-induced leptin release [49]. Ca^2+^ has recently also been shown to participate in the regulation of adiponectin secretion. The ion potently augments cAMP-stimulated adiponectin exocytosis and is also required to maintain sustained adiponectin release over longer time [53,54].

With regard to glucose uptake, a tightly regulated [Ca^2+^]_i_ interval has been shown to be necessary to support the insulin action. A basal [Ca^2+^]_i_ is required for insulin-stimulated glucose uptake [55,56], but excess Ca^2+^ inhibits glucose uptake [55–57].

Evidently, Ca^2+^ is essential for full function of many adipocyte metabolic processes. The source of Ca^2+^ (release from ER stores or influx over the plasma membrane) affecting lipolysis, adipokine secretion or glucose uptake is, however, unclear. In most known endocrine cell types, voltage-dependent Ca^2+^ (Ca_v_) channels are activated upon plasma membrane depolarization leading to influx of Ca^2+^. However, as shown by others [58] and by our own findings (El Hachmane and Olofsson, unpublished), the adipocyte is not an electrically excitable cell type (i.e. electrical excitation does not result in the generation of action potentials). Nonetheless, some studies have suggested the existence of Ca_v_ channels in white adipocytes [59–61], but functional evidence for their presence, at this time, is unavailable. Moreover, the reported adipocyte resting membrane potential of approximately −30 mV [58] suggests that the large majority of Ca_v_s, regardless of subtype, are inactivated under resting conditions [5]. Thus, like in other non-excitatory cell types [11], SOCE likely underlies the main part of Ca^2+^ influx in the white adipocyte. Although SOCE may largely serve to refill intracellular Ca^2+^ stores [25], an interesting prospect is that the locally generated high [Ca^2+^] at the sites of SOCCs specifically acts to regulate the secretion of hormone-containing vesicles that are stored in close vicinity to the plasma membrane [48]. This is suggested in analogy to how Ca_v_s localize to exocytotic sites in other endocrine cell types [62,63]. Of particular interest in this context is the shown cAMP/catecholamine-stimulated adiponectin exocytosis [54,64] since SOCCs have been demonstrated to function in microdomains that generate cAMP [65,66].

### SOCE in metabolic disease

During the last two decades, it has become apparent that disturbed SOCE signalling is connected to a multitude of pathological conditions [67] including metabolic disease [68]. A recent study has pinpointed the importance of STIM1 and SOCE in *Drosophila* adiposity [69]. Preliminary own findings indicate that mRNA levels of *Stim1* are down-regulated in adipocytes isolated from obese and diabetic mice (El Hachmane and Olofsson, unpublished), thus suggesting that adipocyte SOCE may be disturbed also in mammalian metabolic disease. The recently emphasized importance of communication between adipose tissue and the brain [69] further suggests that diabesity-induced alteration of sympathetic nerve innervation of WAT and altered ATP signalling may be part of the metabolic defect. Clearly, the possible connection between impaired STIM1/SOCE activity and diabetes needs to be studied in more detail.

## Abbreviations

ATP: adenosine triphosphate
ER: endoplasmic reticulum
IP_3_: inositol trisphosphate
ORAI1: calcium release-activated calcium channel protein 1
PLC: phospholipase C
qPCR: quantitative polymerase chain reaction
SERCA: sarco/endoplasmic reticulum Ca^2+^-ATPase
siRNA: small interfering RNA
SOCC: store-operate Ca^2+^ channel
SOCE: store-operated Ca^2+^ entry
STIM1: stromal interaction molecule 1
TBS: tris-buffered saline
UTP: uridine-5′-triphosphate
WAT: white adipose tissue
