# Supplementary material for: Extracellular ATP activates store-operated Ca2+ entry in white adipocytes: functional evidence for STIM1 and ORAI1
Source: Biochem J. 2018 Feb 14;475(3):691–704. doi: 10.1042/BCJ20170484 (PMC5813502; doi:10.1042/BCJ20170484)
Supplement: Supplementary Table [file BCJ-475-691-s1.pdf]

Supplementary Table 1

mus musculus Protein symbol	*mus musculus* Gene symbol	Forward primer (5'-3')	Reverse primer (5'-3')	GenBank accession number	Reference (1)
ORAI1	*Orai1*	GCTTTTCCTAGCAGAGGTCGT	AGCGGTAGAAGTGAACAGCAA	NM_175423	
P2RY1	*P2ry1*	GAGGTGCCTTGGTCGGTTG	CGGCAGGTAGTAGAACTGGAA	NM_008772	PrimerBank ID: 6679193a1
P2RY2	*P2ry2*	CTGGAACCCTGGAATAGCACC	CACACCACGCCATAGGACA	NM_008773	PrimerBank ID: 31560661a1
P2RY4	*P2ry4*	ATGACCAGTGCAGACTCCTTG	GAGGCAACAGGATGAACTTGA	NM_020621	PrimerBank ID: 10181172a1
P2RY6	*P2ry6*	GTGAGGATTTCAAGCGACTGC	TCCCCTCTGGCGTAGTTATAGA	NM_183168	PrimerBank ID: 34147203a1
P2RY10	*P2ry10*	GCGTAGGTACGATGTGGGC	GCAAGAATCTGTGCTATTGGCT	NM_172435	PrimerBank ID: 27369579a1
P2RY12	*P2ry12*	CCCTGTGCGTCAGAGACTAC	CAAGCTGTTCGTGATGAGCC	NM_027571	PrimerBank ID: 169790982c1
P2RY13	*P2ry13*	ATGCTCGGGACAATCAACACC	GATGTGGACGAACACCCAGAG	NM_028808	PrimerBank ID: 27229197a1
P2RY14	*P2ry14*	TGGCACAAGGCGTCTAACTAT	GACTTCCTCTTGACGGAGGTG	NM_133200	PrimerBank ID: 56676314c1
STIM1	*Stim1*	TGACAGGGACTGTACTGAAGATG	TATGCCGAGTCAAGAGAGGAG	NM_009287	PrimerBank ID: 145966856c3
TRPC1	*Trpc1*	CTGTGGTATGAAGGGTTGGAA	GAACGTTTGCAAAAGCAAAAA	NM_011643	

1. Primerbank: http://pga.mgh.hardvard.edu/primerbank/

Wang, X., and Seed, B., A PCR primer bank for quantitative gene expression analysis. Nucleic Acids Res, 2003. 31, e154.
